# Effectiveness of conservative instrumentation in root canal disinfection

**DOI:** 10.1007/s00784-023-04929-z

**Published:** 2023-03-03

**Authors:** Sıla Nur Usta, Carmen Solana, Matilde Ruiz-Linares, Pilar Baca, Carmen María Ferrer-Luque, Monica Cabeo, Maria Teresa Arias-Moliz

**Affiliations:** 1 Department of Endodontics, Gulhane Faculty of Dentistry, University of Health Sciences, Etlik, Keçiören, 06018 Ankara Turkey; 2 Department of Stomatology, School of Dentistry, University of Granada, Campus de Cartuja, Colegio Máximo S/N., 18071 Granada, Spain; 3 Instituto de Investigación Biosanitaria Ibs.GRANADA, Granada, Spain; 4 Department of Microbiology, School of Dentistry, University of Granada, Campus de Cartuja, Colegio Máximo S/N., 18071 Granada, Spain

**Keywords:** Conservative instrumentation, Disinfection, Polymicrobial infection, Root canal preparation

## Abstract

**Objectives:**

The impact of conservative instrumentation on the disinfection of root canals with different curvatures has not yet been determined. This ex vivo study aimed to evaluate and compare the effect of conservative instrumentation with TruNatomy (TN) and Rotate and a conventional rotary system, ProTaper Gold (PTG), on root canal disinfection during chemomechanical preparation of straight and curved canals.

**Materials and methods:**

Ninety mandibular molars with straight (*n* = 45) and curved (*n* = 45) mesiobuccal root canals were contaminated with polymicrobial clinical samples. Teeth were divided into three subgroups (*n* = 14) according to the file systems and the curvature. Canals were instrumented with TN, Rotate, and PTG, respectively. Sodium hypochlorite and EDTA were used as irrigants. Intracanal samples were taken before (S1) and after (S2) instrumentation. Six uninfected teeth were used as negative controls. The bacterial reduction between S1 and S2 was measured by ATP assay, flow cytometry, and culture methods. Kruskal–Wallis and ANOVA tests were followed by the Duncan post hoc test (*p* < 0.05).

**Results:**

Bacterial reduction percentages were similar for the three file systems in straight canals (*p* > 0.05). However, PTG showed a lower reduction percentage of intact membrane cells in flow cytometry than TN and Rotate (*p* = 0.036). For the curved canals, no significant differences were obtained (*p* > 0.05).

**Conclusion:**

Conservative instrumentation of straight and curved canals using TN and Rotate files resulted in similar bacterial reduction compared to PTG.

**Clinical relevance:**

The disinfection efficacy of conservative instrumentation is similar to conventional instrumentation in straight and curved root canals.

## Introduction

Apical periodontitis is an inflammatory disease caused by microbial invasion of the root canal and subsequent progress toward extraradicular tissues, leading to bone destruction adjacent to the root [1]. Microorganisms are known to be organized in highly diverse biofilms. The biofilm form helps the bacteria elude the action of antimicrobials and the host response, owing to mechanisms that include the growth of persistent cells, the presence of an extracellular polymeric matrix, and of bacteria in a dormant state [2]. Thus, biofilms are an outstanding challenge in endodontic treatment.

Mechanical instrumentation is the core method for disrupting and reducing bacterial biofilm in the root canal [3]. Yet it cannot completely remove the bacterial load due to the complex anatomy of the root canal, deep bacterial invasion, and physical limitations of the instruments [3, 4]. Additionally, curvatures can limit the cleaning efficiency of instrumentation by leaving canal walls untouched [5]. The outer side of the curvature in the mid-root region and/or the inner side of the curvature in the apical part of the canal may remain uncleaned [6]. Irrigants are therefore needed to enhance disinfection and facilitate the removal of necrotic tissue and debris from difficult-access areas [7].

Minimally invasive endodontics have been gaining attention among efforts to preserve as many dental structures as possible [8]. Instruments with smaller tapers and tip diameters—or made with different geometric designs and metallurgical properties—have been proposed to preserve healthy hard tissue and maintain the strength and function of the tooth [9, 10]. Reducing the preparation size could be advocated for curved canals in view of the lesser undesirable cutting effects and transportation [11]. However, the limited space within the canal and the suggested apical size of 20–25 [12, 13] may jeopardize the disinfection of the apical third in curved canals when conventional irrigation techniques are used. The flow created by the needle is reportedly unable to reach the working length (WL) in minimally shaped canals, regardless of the needle size and the flow rate [14].

Recent years have seen the introduction of several rotary files for conservative root canal shaping. TruNatomy (TN; Dentsply Sirona, Maillefer, Ballaigues, Switzerland) has a slim NiTi wire of 0.8 mm diameter and a square cross-sectioned off-centered design that has been shown to preserve the radicular dentin and maintain the original canal anatomy during instrumentation [12]. Furthermore, TN creates untouched canal walls similar to ProTaper Gold files (PTG, Dentsply Sirona, Maillefer, Ballaigues, Switzerland) [15]. Rotate (VDW, Munich, Germany) is manufactured from blue wire NiTi alloy with an S-shaped sectional design [13]. This file system ensures adequate preparation for narrow and curved root canals given its increased flexibility, the small taper, and the possibility to pre-curve the files [16]. Moreover, Rotate files have higher cyclic fatigue resistance [16] and cause less apical debris extrusion than TN [17].

Although no file system currently available can fully remove bacteria and their by-products from the root canal, the instruments’ mechanical action is still effective for bacterial reduction [18]. To date, we lack studies that assess the disinfection capacity of conservative instrumentation with respect to traditional files. This study aimed to evaluate and compare the effect of conservative instrumentation with TN and Rotate, plus the conventional rotary system PTG, on root canal disinfection during the chemomechanical preparation of straight and curved canals.

## Material and methods

The study protocol was approved by the ethics committee of the university where the study was conducted (no. 1076 CEIH/2020). Ninety mandibular molars with straight (*n* = 45) and curved (*n* = 45) mesiobuccal roots were selected and stored in thymol solution until use. All teeth had closed apexes, no extensive caries, and no previous endodontic treatment. Cone-beam computed tomography (CBCT, PlanmecaProMax 3D; Planmeca, Helsinki, Finland) images of teeth were obtained, and the angle of curvature of the mesiobuccal root canal was measured according to the method of Schneider [19]. Straight canals with a curvature of < 15° and curved canals with curvatures ranging from 20 to 45° were included [20].

Teeth were accessed using a round bur. The WL was determined as 1 mm short of where a #10 K-file (Dentsply Sirona) became visible at the apical foramen. Silicon molds (ZHERMACK elite® HD + , Rovigo, Italy) were made for each tooth to facilitate handling during chemomechanical preparation. The mesiobuccal canals were enlarged up to a #20 K file to ensure space for posterior bacterial contamination. The teeth were treated with 17% ethylenediaminetetraacetic acid (EDTA; Merck, Darmstadt, Germany) for 5 min in an ultrasonic bath to remove the smear layer [21]. The outer surfaces and apexes of the root canals were coated with nail varnish to create a closed-end system. The orifices of the mesiolingual and distal canals were sealed with light-cured resin (R&S Dental Products, Paris, France). Subsequently, teeth and silicone molds were sterilized in an autoclave. The sterility of the dentin was checked by incubating the teeth with Tryptic Soy Broth (TSB; ITW Reagents, Darmstadt, Germany) at 37 °C for 24 h, verifying the absence of turbidity in the medium.

### Microbial sampling and contamination of the specimens

Microbial clinical samples were taken with a file and paper points from canals of teeth with apical periodontitis of volunteers, as previously described [22]. Samples were preserved in saline solution at − 80 °C. The microbial samples were transferred to 5 mL of TSB enriched with 0.005 g/L hemin, 0.001 g/L K vitamin, 5 g/L yeast extract, and 2.5 g/L glucose; they were incubated for 72 h at 37 °C under anaerobic conditions. Following the incubation period, an initial bacterial suspension of 3 × 10^8^ colony-forming units per milliliter (CFUs/mL) was prepared in a turbidimeter (DensiCHECK Plus, bioMerieux, Marcy l’Etoile, France).

The teeth were then immersed in tubes with 5 mL of the polymicrobial suspension for root canal contamination and incubated anaerobically for 21 days. The culture medium was refreshed once a week. After the incubation period, one additional tooth was longitudinally sectioned and processed for observation with scanning electron microscopy (SEM) to confirm biofilm growth on the root canal walls. Briefly, the tooth was first sectioned into two halves, and only the mesial root was kept for SEM visualization. Two vertical grooves in the direction of the curvature were carefully made using a low-speed handpiece with a diamond disk (355514220 HP; Edenta AG, Au/St. Gallen, Switzerland). During this procedure, special care was taken to avoid penetration of the disk in the canal. After obtaining enough space, an enamel chisel was inserted in the grooves, and light pressure was applied in order to separate the two parts. The sample was fixed in 2.5% glutaraldehyde, desiccated, sputter-coated with gold, and viewed under a focused ion beam scanning electron microscope (FIB-SEM; TESCAN AMBER X, Brno, Czech Republic). The rest of the teeth were removed from the tubes and placed in their customized models. The residual culture media in the pulp chamber was removed with a pipette, and the canals were dried with a #20 paper point to eliminate the planktonic bacteria from the root canal space. In order to take the samples, 10 µL of sterile saline solution were added to the mesiobuccal canals. The baseline sample (S1) was taken with a #20 K-file and three #20 paper points. The #20 K-file was placed up to the WL by performing circumferential movements for 30 s. Subsequently, three #20 paper points were likewise inserted up to the WL in the root canal and retained in position for 60 s. The files and paper points were thereafter transferred into Eppendorf tubes containing 500 μL of the enriched TSB, and they were vortexed for 30 s and sonicated for 10 min to recover the bacteria in the culture media.

### Root canal preparation

Teeth with straight and curved canals were divided into three experimental groups (*n* = 14) according to the file system. Group TN included the files #17.02, #20.04, and #26.04; group Rotate, #15.04, #20.05, and #25.04; and group PTG, #18.02, #20.04, #20.07, and #25.08. All files were used according to the manufacturer’s recommendations.

Each mesiobuccal root canal was instrumented to the WL and irrigated with 3 mL 2.5% sodium hypochlorite (NaOCl; Panreac Química SA, Castellar del Vallés, Spain) between files. Irrigation was delivered with a 30-G open-ended needle attached to a 3-mL Luer-lock syringe (DentaFlux, Madrid, Spain). After the instrumentation, the root canals were irrigated with 3 mL of 17% EDTA, followed by a final rinse with 3 mL of 2.5% NaOCl. The NaOCl was inactivated with 1 mL of sodium thiosulfate for 1 min. The canals were dried, and the second samples (S2) were taken with a #25 K-file and three #25 paper points as described above. Six uninfected root canals were used as negative controls, 1 per file system and root canal curvature, following the whole protocol in order to check for the absence of contamination during the laboratory workflow. All these procedures were performed inside a laminar flow chamber (Bio-II-B; Telstar SA, Terrassa, Spain).

According to the program Sample Power 2.0 (SPSS Inc., IBM Corp, Armonk, NY), the sample size of 14 per group allowed for comparison of the quantitative variables between groups with an *α* = 0.05, a power of 80%, and capacity to detect a standardized difference of 1.1 [23].

### Evaluation of the disinfection activity

The evaluation of the presence of microorganisms in S1, S2, and the negative controls was determined by means of the following methods:

#### Adenosine triphosphate (ATP) assay

One hundred microliters of the recovered suspension were added to 100 μL of the BacTiter-Glow reagent (Promega, Madison, WI) and incubated for 5 min [24]. The luminescence produced was measured with a luminometer (GloMax; Promega, Madison, WI). The mean of the signals from the bacterial culture minus the mean of the enriched TSB alone was calculated and expressed as relative light units (RLUs).

#### Flow cytometry analysis

Samples were stained with the LIVE/DEAD BacLight Bacterial Viability kit (Invitrogen, Eugene, OR), which contains Syto 9 that binds to bacteria with intact membranes, and propidium iodide (PI) that labels damaged bacteria. One hundred microliters of the recovered bacterial suspension were stained with 100 μL of a 1:1 Syto 9 and PI mixture for 15 min in the dark. The mixture was then analyzed in a Becton Dickinson FACS Canto II flow cytometer (BD Bioscience, San Jose, CA). The results were analyzed using the software FACSDiva Version 6.1.3 (Becton, Dickinson) to derive a graph of two-dimensional points representing the different cell populations within the sample. Values of membrane-intact bacteria (stained with Syto 9) were thereafter analyzed.

#### Culture method

Ten microliter aliquots of serial dilutions (10^−1^ − 10^−5^) from the recovered suspensions were plated and incubated under anaerobic conditions for 72 h at 37 °C. The number of CFUs/mL was then calculated.

### Statistical analysis

The ATP assay and flow cytometry results were respectively expressed as the reduction percentages (*P*) of the RLUs and intact membrane cells of S2 with respect to S1, previously subjecting the data to the logit transformation: Ln (*P*/(1 − *P*)). CFU data were expressed as Log10 (CFUs + 1). The logarithmic reduction was also calculated. The Shapiro–Wilk test served to check the normality of the transformed variables. Multiple comparisons of variables that did not follow a normal distribution were performed by means of the Kruskal–Wallis test, while an ANOVA test was used for normal variables. In the event of differences in the ANOVA test, pair-by-pair comparisons by the Duncan post hoc test were performed after checking that the variances were similar. The level of significance was set at *p* < 0.05. Statistical analyses were performed using SPSS 20.0 software.

## Results

**Fig. 1:**
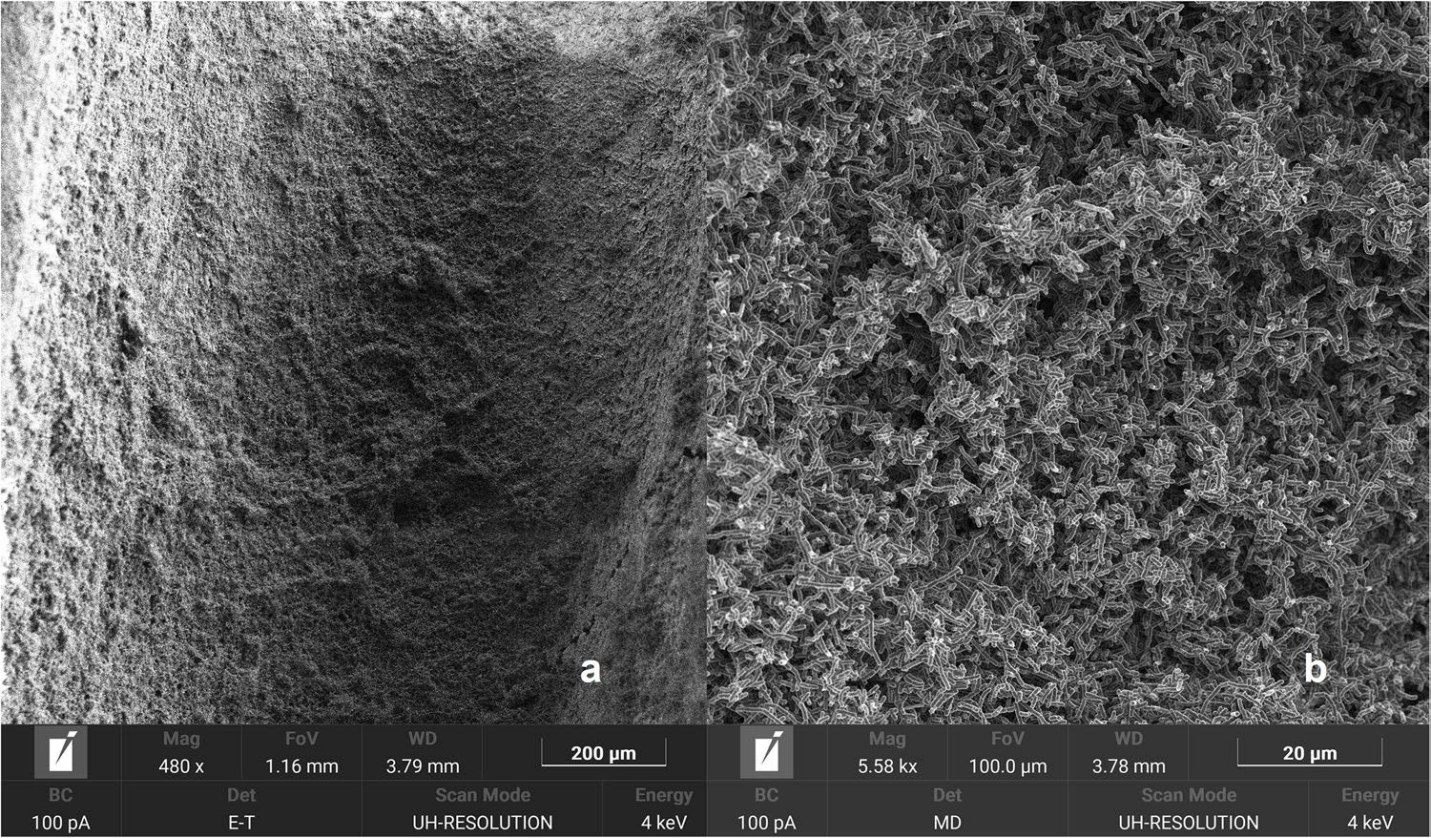
Representative focused ion beam scanning electron microscope (FIB-SEM) microphotograph of the root canal contamination. Dense biofilm is growing on the root canal walls at 480X (**a**) and 5.58KX (**b**)

The sterility and negative controls gave negative results under all three evaluation methods. There were no differences in the results of the S1 samples in the three tests in straight and curved canals (*p* > 0.05), which indicates that the samples were homogenously contaminated. Figure 1 shows a representative FIB-SEM microphotograph of the biofilm grown on the root canal walls.

In the straight canals, the percentages of reduction of the RLUs and the logarithmic reductions of the CFUs obtained with the three file systems were statistically similar (*p* > 0.05). Differences were, however, observed in the reduction percentages of intact membrane cells obtained by flow cytometry—significantly lower in the PTG group than for TN and Rotate (*p* = 0.036). Results of the effect of the three systems on the microorganisms in straight canals are shown in Table 1. According to the three assays, no differences were observed for any file system in the curved canals, as presented in Table 2 (*p* > 0.05).

**Table 1:** Results of the antimicrobial activity of the three file systems in straight canals evaluated by the ATP assay, flow cytometry, and culture assay (*n* = 14/group). Mean (standard deviation)

	TruNatomy	Protaper Gold	Rotate	Comparisons *p-*value
ATP assay				
S1 (RLUs)	47,083.86 (29,230.12)	44,232.79 (17,586.32)	62,158.43 (33,159.25)	0.302*
Reduction % in S2	88.84 (24.02)	96.88 (1.54)	96.60 (3.02)	0.185*
Cytometry				
S1 (live cells)	16,234.85 (10,904.43)	14,014.92 (3948.55)	20,554.50 (9001.16)	0.128*
Reduction % in S2	86.37 (15.87)^a^	78.13 (14.07)^b^	89.77 (5.37)^a^	0.036**
CFUs assay				
S1 (Log_10_)	6.23 (0.39)	6.06 (0.49)	6.19 (0.27)	0.521**
Log reduction (S2-S1)	3.18 (0.56)	3.41 (0.61)	3.07 (0.39)	0.249**

*S1* basal sample, *S2* sample after treatment, *RLUs* relative light units

^*^Multiple comparisons by the Kruskal–Wallis test

^**^Multiple comparisons by the ANOVA test

Read horizontally, the same letters show no statistical differences by the Duncan post hoc test

**Table 2:** Results of the antimicrobial activity of the three file systems in curved canals evaluated by the ATP assay, flow cytometry, and culture assay (*n* = 14/group). Mean (standard deviation)

	TruNatomy	Protaper Gold	Rotate	Comparisons *p-*value
ATP assay				
S1 (RLUs)	179,675.92 (146,223.35)	133,542.35 (63,834.29)	177,522.35 (121,601.65)	0.583*
Reduction % in S2	99.49 (0.52)	99.34 (0.52)	99.6 (0.19)	0.379**
Cytometry				
S1 (live cells)	17,617.50 (3372.08)	19,011.07 (3386.05)	18,748.28 (3426.08)	0.354*
Reduction % in S2	58.62 (10.56)	63.27 (6.25)	60.82 (12.70)	0.292**
CFUs assay				
S1 (Log_10_)	6.12 (0.31)	6.03 (0.37)	6.34 (0.16)	0.056*
Log reduction (S2-S1)	4.01 (1.25)	3.55 (1.23)	3.61 (1.25)	0.363*

*S1* basal sample, *S2* sample after treatment, *RLUs* relative light units

^*^Multiple comparisons by the Kruskal–Wallis test

^**^Multiple comparisons by the ANOVA test

## Discussion

Minimally invasive endodontics are intended to preserve the maximum amount of root canal dentin [8]. Even though conservative cavity access and root canal preparation with small apical sizes and tapers may not significantly impact the treatment outcome in teeth with vital pulps [25], they might compromise canal disinfection in teeth with apical periodontitis [26], as irrigants could have difficulty in penetrating to the WL, especially in curved root canals [14]. To our knowledge, no study has evaluated the effect of chemomechanical preparation with reduced-taper files on root canal disinfection in straight and curved canals.

Clinical samples were taken to contaminate the canals, so as to create natural multispecies biofilms that resemble in vivo root canal biofilms more closely than single-species biofilms [27]. Three different methods were selected to evaluate the disinfection efficacy since there is no gold-standard method [28]. Although the culture technique is most widely used for bacterial detection, it cannot detect viable but non-culturable (VBNC) bacteria, thus underestimating the number of bacteria in multispecies biofilms. On the contrary, ATP detects the community’s metabolic activity, including viable and VBNC cells [29]. Flow cytometry was furthermore included because it classifies the cells according to the state of the membrane, so that intact-membrane bacteria could be considered alive and damaged cells as dead. One limitation of this technique is that cells with intact membranes can be metabolically inactive, thus dead, whereas cells with damaged membranes may still be alive, leading to false results [30].

The anatomy analysis and the measurement of root canal curvature were performed using CBCT instead of micro-computed tomography (micro-CT), since it is a reliable and non-destructive method for evaluating root canal morphology [31]. CBCT scans can be used to characterize the majority of root canal configurations and shapes and provide specific measurements such as angles for root canal curvature [32–34]. Additionally, CBCT scans of extracted teeth constitute a cost-effective 3D method yielding rapid data presentation compared with micro-CT. Micro-CT, in turn, provides higher-quality images with improved resolution [35, 36], though it is time-consuming and hinders the possibility of obtaining a large number of samples [37, 38].

In this study, conservative root canal shaping with TN and Rotate reduced the microorganisms to a degree similar to PTG, regardless of the curvature. The higher taper in the PTG group did not affect the root canal cleanliness, a finding in line with previous reports of similar debris [9] and bacterial reduction [39] using different tapers. One study did find that increasing the taper from 4 to 8% caused a significant difference in the number of residual bacteria [40]. The mono-species biofilm, the different types of teeth and file systems, and the evaluation method may explain such contradictory results. Interestingly, this study observed that the reduction percentage of membrane-intact cells obtained by flow cytometry in the PTG group was statistically lower than for TN and Rotate in straight canals. One explanation is that some membrane-intact bacteria in the PTG group could be non-culturable and non-active metabolically, hence most probably detected as dead in the ATP and CFU assays, leading to the lower reduction percentage in the PTG group obtained by flow cytometry [41]. Otherwise, an overall good correlation between the CFUs and RLUs was observed, which is in line with previous studies [42].

The absence of different results among the three files could be attributed to the fact that the areas touched by the instruments might be similar, given that instruments tend to remain centered in the root canal. Therefore, increasing the instruments’ taper may increase the volume of the root canals, but the unprepared areas could still remain in the irregular regions [9]. Additionally, the cross-sectioned off-centered design of the TN files—unlike the conventional concentric design in the PTG—creates a snakelike motion that allows the instrument to touch more canal walls even though it has smaller dimensions. This snakelike motion has been associated with an increase in the space for removing pulp remnants and debris [15]. Finally, even though PTG presents larger tapers than TN and Rotate, the apical size is the same, so similar apical cleanliness is expected [9, 15]. Taper may be less important for irrigant penetration, especially in the apical third [43].

The results of this study also support the importance of irrigation in treating root canal infection. The irrigation protocol selected is the most accepted one [44]. NaOCl is a strong antibiofilm irrigant with dissolution properties, and EDTA reduces the smear layer and debris. Furthermore, final irrigation with NaOCl was included as it may enhance disinfection [44]. The antimicrobial efficacy of this protocol might have compensated for the limitations owing to smaller preparations.

The disinfection of the apical area of the teeth with curved canals could be considered a matter of anatomical challenges [5]. Interestingly though, the curvature did not appear to influence the disinfection efficacy of the file systems in this study. The stress generated by the instruments in curved canals has been found to remove more dentin apically on the curvature’s outer side [45]. Accordingly, higher strains between instruments and canal walls in curved canals than in straight ones would explain this result [45].

In the wake of our findings, several limitations should be addressed. Firstly, syringe irrigation using 30-G open- and 31-G close-ended needles can be compromised in minimally shaped canals with less than 30.06 apical size, as the irrigant might not be delivered up to the working length [14]. To overcome this limitation, open-ended needles were used instead of closed ones, to create a jet toward the apex and increase the irrigant replacement [43, 44]. Secondly, the sampling technique using a file and a paper point recovers mainly planktonic bacteria from the main root canal or loosely adhered to the wall [46] and cannot provide information on the location of the bacteria. Finally, this study is of a laboratory nature, reflecting only two root canal configurations (straight and curved). Further studies are needed to evaluate the effectiveness of conservative instrumentation in teeth having different root canal configurations and at different sites.

In conclusion, conservative instrumentation of straight and curved root canals using TN and Rotate file systems resulted in similar bacterial reduction when compared to PTG during chemomechanical preparation. None of the instruments were able to create a bacteria-free canal system.

## Data Availability

The data that support the findings of this study are available from the corresponding author upon reasonable request.
